# Fungal Mitogenomes: Relevant Features to Planning Plant Disease Management

**DOI:** 10.3389/fmicb.2020.00978

**Published:** 2020-05-29

**Authors:** Rocio Medina, Mario Emilio Ernesto Franco, Laura Cecilia Bartel, Virginia Martinez Alcántara, Mario Carlos Nazareno Saparrat, Pedro Alberto Balatti

**Affiliations:** ^1^Centro de Investigaciones de Fitopatología, Comisión de Investigaciones Científicas de la Provincia de Buenos Aires (CIDEFI-CICPBA), Facultad de Ciencias Agrarias y Forestales, Universidad Nacional de La Plata, La Plata, Argentina; ^2^Department of Biosystems Engineering, The University of Arizona, Tucson, AZ, United States; ^3^Cátedra de Microbiología Agrícola, Facultad de Ciencias Agrarias y Forestales, Universidad Nacional de La Plata, La Plata, Argentina; ^4^Instituto de Fisiología Vegetal (INFIVE), Consejo Nacional de Investigaciones Científicas y Técnicas (CONICET), Universidad Nacional de La Plata, La Plata, Argentina

**Keywords:** fungal mitogenome, plant disease management, mobile elements, pathogens, virulence, pathogenesis, fungal interactions

## Abstract

Mitochondrial genomes (mt-genomes) are characterized by a distinct codon usage and their autonomous replication. Mt-genomes encode highly conserved genes (mt-genes), like proteins involved in electron transport and oxidative phosphorylation but they also carry highly variable regions that are in part responsible for their high plasticity. The degree of conservation of their genes is such that they allow the establishment of phylogenetic relationships even across distantly related species. Here, we describe the mechanisms that generate changes along mt-genomes, which play key roles at enlarging the ability of fungi to adapt to changing environments. Within mt-genomes of fungal pathogens, there are dispensable as well as indispensable genes for survival, virulence and/or pathogenicity. We also describe the different complexes or mechanisms targeted by fungicides, thus addressing a relevant issue regarding disease management. Despite the controversial origin and evolution of fungal mt-genomes, the intrinsic mechanisms and molecular biology involved in their evolution will help to understand, at the molecular level, the strategies for fungal disease management.

## Origin and Evolution of Mitochondria

**FIGURE 1:**
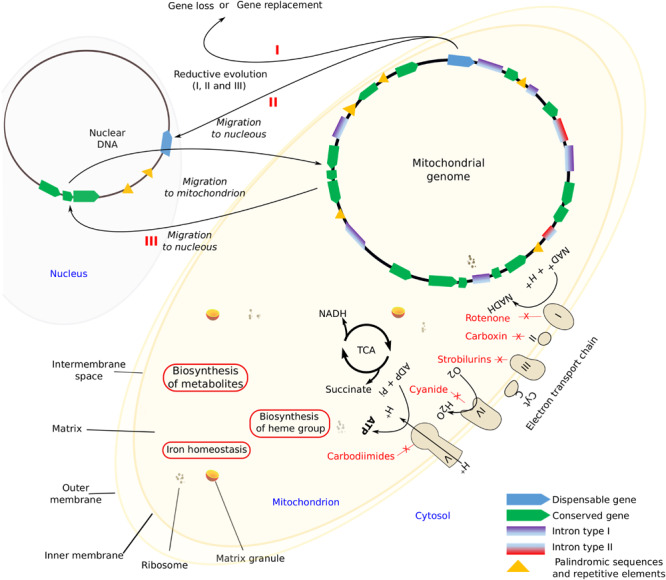
Mitochondrial components and the main features. I, II, and III (in red) schematic processes that lead to the size reduction of mitochondrial genome by gene loss.

Mitochondria are highly dynamic organelles with a double-membrane that carries the respiratory complex that generates adenosine triphosphate (ATP), the chemical energy currency of cells (Roger et al., 2017). Several processes occur within mitochondria, like the aerobic citric acid cycle (TCA), electron transport system that results in the synthesis of ATP, the biosynthesis of metabolites like amino acids, the heme group, and the Fe-S centers as well as reactions that regulate cellular iron homeostasis (Verma et al., 2018; Figure 1). In addition to this, mitochondria play crucial roles in resistance to microbial antagonists as well as in host pathogen interactions (Rudel et al., 2010; Hadwiger and Polashock, 2013).

In 1970, Margulis hypothesized that eukaryotic organelles like mitochondria evolved from endosymbiotic bacteria (Margulis, 1970). The development of biotechnological tools together with the development of phylogenetic analysis software allowed researchers to focus their studies on the origin of mitochondria that consisted in the full integration of an alphaproteobacterium into a host cell that was related to Asgard, an Archaea that produces an organelle capable of aerobic respiration as well as other biochemical functions that also occur in mitochondria (Szklarczyk and Huynen, 2010; Wang and Wu, 2014; Gray, 2015). Also, several researchers suggest that mitochondria originated in subsequent temporary events, essentially at the same time as the nucleus of eukaryotic cells rather than from a common protomitochondrial ancestor (Gray and Spencer, 1996; Andersson et al., 1998; Gray et al., 1999). Eukaryotic evolution started with the last eukaryotic common ancestor (LECA) (Koonin, 2010; Klöpper et al., 2012). One of the earliest branches of eukaryotes is a population of amitochondriate organisms that diverged away from the mainline before the advent of mitochondria (Martin and Müller, 1998; Müller et al., 2012). Recently, a study considered that LECA might not be a pangenomic population (O’Malley et al., 2019). So, an ancestral mitochondrial genome (mt-genome) might be defined as the one that retained vestiges of its eubacterial ancestry (Gray et al., 1999). Regarding mt-genomes, a study based on 14 mitochondrial genes demonstrated that *Ascomycetes* started to diverge earlier than *Basidiomycetes* (Wang et al., 2019). Within Dikarya, in general *Basidiomycetes* mt-genomes are highly variable in gene order compared to *Ascomycetes*. Furthermore, while in *Basidiomycetes* genes are usually encoded on both strands, in *Ascomycetes* they are in only one.

Regarding evolution, most fungal mt-genomes are characterized by gene loss (Spanu et al., 2010), marked divergence in ribosomal DNA and rRNA structures (Lang et al., 1999; Petrov et al., 2018), adoption of a highly biased codon usage strategy in protein genes (Gray et al., 1999), elimination of certain codons (Nedelcu and Lee, 1998), and also the introduction of non-standard codon assignments (Sengupta et al., 2007). Interestingly, evolution within genes encoding typical mitochondrial proteins was observed in nuclear genomes of amitochondriate protists (Timmis et al., 2004). Furthermore, hydrogenosomes, organelles that generate ATP anaerobically and are characteristic of obligate anaerobic fungi that live within the gut of mammals, known as Neocallimastigales, contained mitochondrial proteins (Hackstein et al., 2019). Mitosomes are highly reduced and cryptic organelles of Microsporidia, that do not produce ATP. They are related either to the iron-sulfur cluster assembly (Burri et al., 2006; Müller et al., 2012) or linked to mitochondria-related organelles in anaerobic and microaerophilic lineages (Burki, 2016). The gene loss that led to a reductive mitochondrial evolution and mitochondrion related organelles is neither a result of functional redundancy nor of gene selection, it is mostly a structural adaptation. Proof of this is that hydrogenosomes and mitochondria have a common evolutionary origin and that amitochondriate eukaryotes once had mitochondria (Henze et al., 1995; Müller, 1997; Doolittle, 1998).

Evolutionary divergent processes might be relatively rapid and might lead to an extensive loss of mt-genes. Adams and Palmer (2003) established three alternative reductive processes, namely: the loss of non-essential functions, substitution, and gene transference to the nucleus (Figure 1). Franco et al. (2017) found that in *Stemphylium lycopersici* (Pleosporales, Ascomycota) the widely conserved mt-genes *atp8* and *atp9* migrated to the nucleus. Such transference might transform a non-functional protein into a functional one, otherwise pseudogenization may occur, whereas the opposite, that is, the replacement of mt-genes by nuclear ones of similar function can also occur. However, it is possible that, the non-standard genetic mitochondrial code is probably preventing further gene transfers of functional proteins (Boore, 1999). In any case, all these processes lead to a size reduction of mt-genomes.

## Characteristics of Fungal Mitogenomes

**FIGURE 2:**
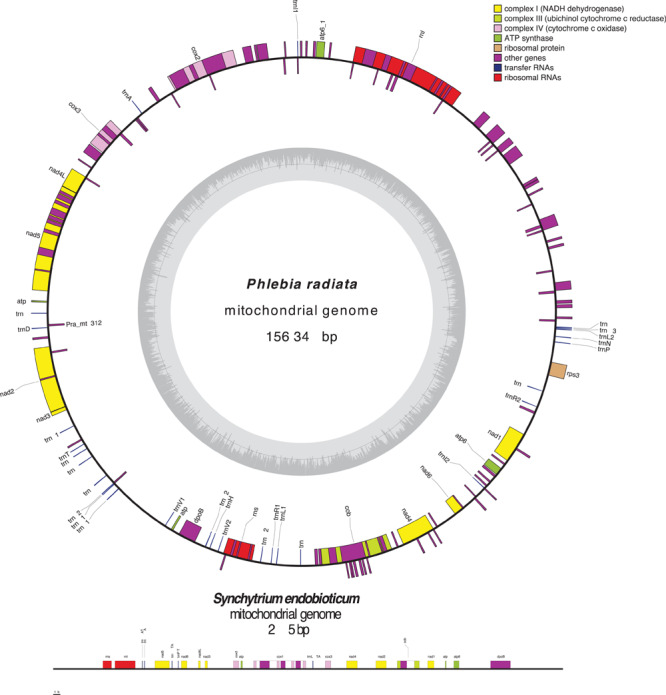
Array and orientation of core genes, the untranslated genes of small and large ribosomal RNA subunits, and the AT/GC content of the circular mt-genome of *Phlebia radiata* (NC_020148, Basidiomycota) and the linear mt-genome of *Synchytrium endobioticum* (NC_042370, Chytridiomycota). Figure was made using OrganellarGenomeDRAW (OGDRAW) version 1.3.1 (Greiner et al., 2019).

Fungal mt-genomes are highly diverse in conformation and size (Lang et al., 2007; Aguileta et al., 2014), gene content, order, and expression (Adams and Palmer, 2003; Shao et al., 2003). Most of them exhibit a circular-mapping topology, like a typical bacterial genome, however, some are linear concatemeric structures (Figure 2), that are most likely products of a rolling-circle mechanism of replication, which is frequent in organisms with linear mt-genomes (Maleszka et al., 1991; Maleszka and Clark-Walker, 1992; Bendich, 1993, 1996; Ling and Shibata, 2002; Hausner, 2003; Salavirta et al., 2014). Within the phylum Chytridiomycota, *Synchytrium endobioticum*, an obligate biotrophic pathogen of potato, and *Hyaloraphidium curvatum* have both linear mt-genomes, though the later one with terminal inverted repeats (Forget et al., 2002; van de Vossenberg et al., 2018). This raises a question as to whether linearity or circularization is related to evolution or if this is a step within evolution that separated organisms in different evolutionary branches.

Fungal mt-genomes are characterized by a high AT-content and an ample range of genome sizes. *Spizellomyces punctatus* (Chytridiomycota), a saprotrophic representative that is also found in association with a range of mycorrhizal fungi, mildews, plants, and soil nematodes (Russ et al., 2016), has a mitogenome composed of three chromosomes, and only one of them has 1136 bp. The mt-genome encodes only 8 tRNAs, while they normally carry 24–25 tRNAs (Laforest et al., 1997). On the other extreme, the mitogenome of *Morchella importuna* (Ascomycota), the largest one known, has 272,238 bp long and encodes a set of 32 different tRNAs (Liu et al., 2020). The size of the mt-genome is probably the result of (i) length and organization of intergenic regions; (ii) presence of introns (type I and II) of various sizes and numbers (Burger et al., 2003); (iii) intron-encoded open reading frames and AT-rich intergenic spacers (Hausner, 2003); (iv) palindromic sequences scattered throughout AT-rich intergenic spacers (Yin et al., 1981); (v) tandem-repeat arrays; (vi) stem-loop motifs (Paquin and Lang, 1996); (vii) ultra-short elements (Hausner, 2003); and (viii) evolutionary divergent processes.

Group I and II introns and maybe other introns as well are self-splicing DNA sequences that play a major role in genome evolution (Schuster et al., 2017). They are distinguished by their splicing mechanisms and secondary structures. Frequently, they contain in their loop regions open reading frames (ORFs) that encode different site-specific homing endonuclease genes (HEGs, Schäfer, 2003). Most group I introns include HEGs, with a conserved single or double LAGLIDADG amino-acid sequence motif. In contrast, group II introns encode mostly reverse transcriptase-like (RT) ORFs (Hausner, 2012). While the latter group of introns seems to thrive especially in plants, introns type I mainly occur within fungi (Santamaria et al., 2009; Férandon et al., 2010; Hafez and Hausner, 2012; Li et al., 2015). Other mobile elements identified in fungal mt-genomes are plasmid and plasmid-like DNA sequences. While the former ones are linear or circular DNA with no homology with the mt-genome, the latter are covalently closed DNA sequences homologous to regions within the mt-genome (Hausner, 2003). Plasmid elements usually have been associated with nuclear or mitochondrial mutations (Férandon et al., 2010; Beaudet et al., 2013) and provoke changes in the mt-genome architecture. Mt-genomes also contain palindromic sequences scattered throughout AT-rich intergenic spacers that frequently form long highly stable hairpin structures, where DNA recombination (Hausner, 2003) or replication or transcription initiation occur that might be the primary sites for processing transcripts (Gross et al., 1989a, b). Also, mt- genomes contain ultra-short elements (MUSEs) that are highly repeated recombinant sequences that are associated with excision and amplification of short mitochondrial segments. These processes often occur during the phenomenon of degenerative senescence in *Podospora anserina* (Koll et al., 1996) and consist in a progressive loss of growth potential culminating in hyphal death. MUSEs are highly invasive and contribute to the mt-genome evolution. Additionally, mt-genomes contain introns that have been associated with mt-genome defects (Hausner, 2012), rearrangements (Wu et al., 2015), and diversity of sequences flanking introns (Repar and Warnecke, 2017).

**FIGURE 3:**
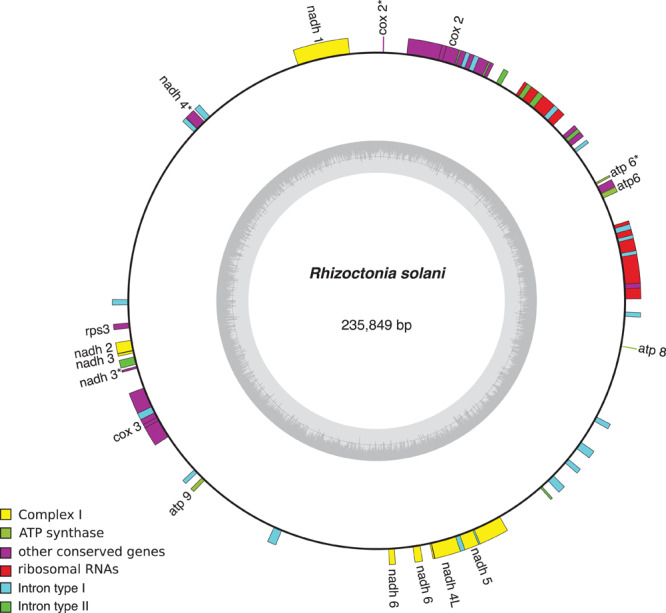
*Rhizoctonia solani* mitochondrial genome and the type and number of introns present. The complete mt-genome is 235,849 bp long and encodes 15 conserved genes (showed in yellow). Group I introns are in red whereas group II are in blue. NCBI Reference Sequence: NC_021436.1. Figure was made using OrganellarGenomeDRAW (OGDRAW) version 1.3.1 (Greiner et al., 2019).

Mobile elements play a key role in the expansion of fungal mt-genomes. Organisms like *Endoconidiophora resinifera*, (Microascales, Ascomycota), a fungus associated with blue-stain on sapwood, and *Phlebia radiata* (Polyporales, Basidiomycota), a fungus that colonizes wood associated with white-rots, have large mitogenomes >220,000 bp and 156,348 bp, respectively. While *E. resinifera* has a large number of intron insertions within coding sequences of the mt-genome (Zubaer et al., 2018), *P. radiata* has many introns as well as intergenic regions that account for 80% of mt-genome (Salavirta et al., 2014). Both, introns and short repetitive sequences, are dispersed and sometimes overlap and/or interrupt conserved genes (*cox1, cox2, cox3*; *cob*; *nad1, nad2, nad4, nad4L, nad5*; *rnl* and *rns*). Another example is *Rhizoctonia solani* (Cantharellales, Basidiomycota), a potato pathogen, whose mt-genome, that is one of the largest one within filamentous fungi, accumulated introns and repeated sequences (Figure 3; Losada et al., 2014). It appears that mobile elements like introns and repetitive sequences are responsible for mt-genome expansion, mostly due to the accumulation of repeated sequences and AT-rich intergenic spacers, mobile elements, and introns (Hausner, 2012). Even within a fungal genus, the length of non-coding intergenic regions in the mt-genome can vary considerably and introns might be splitting genes (Salavirta et al., 2014). Future studies should be aimed at studying if evolution is mostly related to the effect repetitive or mobile elements have on gaining abilities or with the identification of unknown capacities.

Mt-genomes have more unusual gene organizations, like gene fusions (Burger et al., 2003), and order that is poorly conserved due to the loss, transfer and/or gene rearrangements that occur due to insertions, deletions or recombination, that follow specific rules owing to the multicopy character of mt-genomes. Still, the ancestral arrangement in some mt-genomes is quite evident (Paquin and Lang, 1996). Franco et al. (2017) while studying the systematic of the subphylum Pezizomycotina using the amino acid sequences of 12 conserved mt-genes that code for proteins of the oxidative phosphorylation and electron transport system showed that Pezizomycotina is a monophyletic subphylum, which was in agreement with the nuclear phylogeny described by Spatafora et al. (2006). In a way, this shows the importance conservation has within the mt-genome but at the same time, that plasticity is essential since it allows the occurrence of changes, evading in this way quite frequent lethal phenotypes that might arise along the process.

Fungal mt-genomes usually harbor 14 core-genes encoding proteins of the electron transport and oxidative phosphorylation pathway, including the apocytochrome b (*cob*), 3 subunits of the cytochrome c oxidase (*cox1*, *cox2*, *cox3*), 7 subunits of the reduced nicotinamide adenine dinucleotide ubiquinone oxidoreductase (*nad1*, *nad2*, *nad3*, *nad4*, *nad4L*, *nad5*, and *nad6*), and 3 subunits of the ATP synthase (*atp6*, *atp8*, *atp9*) (Figure 2). Untranslated genes of the small and large ribosomal RNA (rRNA) subunits (*rns* and *rnl*, respectively) and a set of transfer RNA (tRNA) genes also are part of the mt-genome. Other genes occasionally found are those encoding the ribosomal protein S3 (RPS3, coded by *rps3*), a component of the 40S subunit that plays a critical role in the initiation of protein translation and the RNA subunit of the mitochondrial RNase P (*rnpB*; Adams and Palmer, 2003; Férandon et al., 2013; Franco et al., 2017; Table 1). Additionally, Paquin et al. (1997) showed that fungal mitochondrial tRNA genes were processed suggesting that such mismatch repair prevents tRNA processing by RNAse P and/or other enzymes. In line with this, in *S. cerevisiae* homing endonucleases, encoded within the group I intron, can generate a double-stranded break, which is a key step at the beginning of the repair process that involves unidirectional homologous recombination (Dujon et al., 1989). The ribosomal protein S3 (RPS3) is a multi-functional protein encoded within the group I intron or by a free-standing gene sequence (Zimmer et al., 1991; Wool, 1996). RPS3 is involved in DNA repair due to their DNA endonuclease activity (Kim et al., 1995), a function dependent on the level of phosphorylation by PKCδ, which phosphorylates serine 6 and threonine 221 in response to DNA-damaging agents (Kim et al., 2009). RPS3 also plays a role in cell signaling, apoptosis/survival, and transcriptional regulation (Kim et al., 2013). Interestingly, RPS3 is highly conserved among fungal mt-genomes and can be translocated into the nuclear genome through a “cycling” mechanism (Wai et al., 2019). DNA maintenance and/or repair mechanisms in fungal mt-genomes should be deepened to understand their biological significance and to develop new strategies to manage the attack of phytopathogenic fungi.

**TABLE 1:** Conserved genes encoded in the fungal mtDNA.

	**gene**	**Biological process**
apocytochrome *b*	*cob*	Electron transport
cytochrome c oxidase	*cox1, cox2, cox3*	Oxidative phosphorylation
reduces nicotinamide adenine dinucleotide ubiquinone oxidoreductase	*nad1, nad2, nad3, nad4, nad4L, nad5, and nad6*	
ATP synthase	*atp6, atp8, atp9*	ATP synthesis
r RNA	*rns, rnl*	Small and large ribosomal RNA
tRNA	*tRNA*	Transfer RNA
Ribosomal protein	*rps3*	Component of the 40S subunit, initiation of protein translation, cell signaling, apoptosis/survival, transcriptional regulation, and DNA repair
RNAseP	*rnpB*	Subunit of the mitochondrial RNAsP

Hence, the mt-genome uses particular genetic mechanisms of recombination mediated by mobile elements. Furthermore, mt-genes can migrate to the nucleus or be lost after a short time or they might be transiently expressed, unless they are stably integrated into the mt-genome. Besides, point mutations may inactivate genes, and this inactivation may be transient due to the mitochondrial mismatch repair. In summary, it is necessary to know the process that governs the dynamics of the mt-genome as well as its effect on the genetic environment it creates within the mt-genome and its impact in nature.

## Conservation Vs. Plasticity

Fungal mt-genomes generally show the right trade-off between conservation and plasticity that makes them suitable tools for phylogenetic, population, and comparative analyses. On the one hand, they harbor a set of genes that code for proteins involved in the electron transport-oxidative phosphorylation system. Given their vital role in cell physiology, such genes are highly conserved even across distantly related species. For this reason, the use of amino acid sequences of core mt-genes has become a standard practice in mitochondrial-based phylogeny (Burger et al., 2003; Kouvelis et al., 2005; Pantou et al., 2006). In fact, they proved to be useful in solving many unclear phylogenies (Bullerwell et al., 2003; van de Sande, 2012). On the other hand, fungal mt-genomes frequently contain repetitive and mobile elements within introns and intergenic regions between conserved genes, that usually are highly polymorphic. These characteristics make them useful tools to study diversity among or within populations (Formey et al., 2012; Joardar et al., 2012; Zhang et al., 2015). Moreover, the relatively small size, circular-mapping topology, and multi-copy nature of the mt-DNA simplify not only the sequencing but also their assembly, enabling the implementation of robust comparative analyses.

The mt-genome of *Tolypocladium inflatum* (Hypocreales, Ascomycota) is one of the smallest ones carrying a limited number of highly conserved key genes among organisms with similar lifestyles. Such compact mt-genome is the result of several non-typical repetitive elements suggesting that gene transfer occurred from the nucleus to the mitochondria. Interestingly, this mt-genome has a lower level of intraspecific variation compared to the nucleus (Zhang et al., 2017; Muthabathula et al., 2019). In line with this, the mt-genome of *Hypomyces aurantius*, a mycoparasite that causes cobweb disease, share several features like introns and gene arrangement with the mt-genomes of other members of the Hypocreales order, except for *Acremonium chrysogenum* and *Acremonium implicatum*. Still, the mt-genome of *H. aurantius* was larger due to the presence of 17 introns in six conserved genes, *cox1*, *rnl*, *cob*, *atp6*, *cox3*, and *cox2* (Deng et al., 2016). The mt-genome of related species such as *S. endobioticum* and *S. microbalum* (Synchytriales) presented polymorphic mt-genomes with no conserved organization and/or orientation of genes even though they are related (van de Vossenberg et al., 2018) and recently suffered a process of linearization and expansion. Another example of mt-genome plasticity is the mt-genome of *Laetiporus sulphureus* (Polyporales, Basidiomycota) that carry 38 coding sequences and 2 rRNA and 25 tRNA genes most probably due to horizontal transfer. As a consequence of this, tandem repeats accumulated in the mt-genome, leading this to its expansion, which has been crucial to proceed to differentiate species or study evolution (Li et al., 2018a). Interestingly, the GC content of the mt-genome of *L. sulphureus* was, compared to other species of Polyporales, the highest one. In this genome, only *cox1*, *cob*, and *rnl* appeared as potentially useful molecular markers since the other conserved genes were exposed to a purifying selection. So, the degree of gene conservation and ordination within fungal mt-genome seemed to be dependent upon the fungal group studied. In this regard, an exhaustive analysis should be performed before selecting the phylogenetic molecular markers.

Regarding the mt-genome variation within isolates of the same species, results are controversial. While strains of the arbuscular mycorrhizal fungus, *Rhizophagus irregularis* (Glomerales, Glomeromycota), as well as of *T. inflatum* (Hypocreales) presented single-nucleotide polymorphism within the nuclear genome, the mt-genome presented not even one (Formey et al., 2012; Zhang et al., 2017). Besides, closely related yeast species presented a lower level of mitochondrial nucleotide diversity than the orthologous nuclear genes (Freel et al., 2014). On average, mt-genomes had a higher sequence identity than nuclear coding sequences, which is probably due to a lower rate of mitochondrial evolution (Gaillardin et al., 2012). Conversely, the mt-genome of four closely related crop pathogens of the genus *Rhynchosporium* (Heliotiales, Ascomycota) presented 77 times higher variation than the nuclear genomes (Torriani et al., 2014). The analysis of the mt-genome of representatives of the *Hymenochaetales* (Basidiomycota) indicated that evolution is usually lineage-specific, though chimeric mitotypes are frequent, suggesting that the evolutionary processes shaping mitogenomes are similar to those described in other *basidiomycetes* (Lee et al., 2019).

**FIGURE 4:**
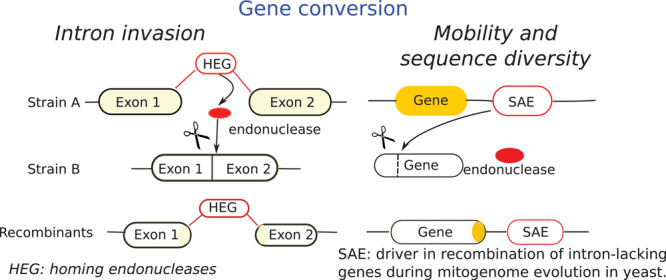
Scheme of gene conversion through intron invasion and mobility and diversification adapted from Wu and Hao (2019). SAE mechanisms, known as intronless homing is compared with intron homing mechanism (HEG). Intron homing is facilitated by intron-encoded endonuclease and occurs into homologous intron-minus genes. SAE occurs in adjacent intron-lacking genes and facilitate gene conversion of their neighboring genes. The horizontal lines indicate endonuclease site.

Mobile elements within fungal mt-genome may be potential tools for the genetic engineering of fungi. Introns, containing only genes coding endonucleases, facilitate recombination of intron-containing genes since they occur due to the activity of an intron homing mechanism that facilitates recombination, such regions might turn out to be recombination hotspots (Wu and Hao, 2019). Stand-alone endonucleases (SAEs) are other mobile elements of fungal mt-genomes (Beaudet et al., 2013; Franco et al., 2017). Wu and Hao (2019) studied the mechanisms of recombination in mitochondria and found that SAEs mediate gene conversion of adjacent intron-lacking genes. In line with this, they proposed the mechanism described in Figure 4.

The diverse size as well as the structure of fungal mt-genomes is an area that deserves special attention. The mt-genome of the acaropathogenic fungus *Hirsutella thompsonii* (Hypocreales) ranges in size between 60.3 and 66.4 kb and such differences were due to the presence of a variable number of introns. Among the 15 intron *loci* identified, more likely four were acquired by horizontal transfer from other fungi, suggesting this that their genomes are highly dynamic (Wang et al., 2018). On the other hand, the mt-genome of isolates of *Annulohypoxylon stygium* (Xylariales, Ascomycota), a cellulolytic fungus, ranged between 132 and 147 kb but they code for the same sets of conserved protein, tRNA and rRNA genes. Furthermore, even though they shared the arrangement and orientation, the content and distribution of introns are highly variable, which can be explained by movements, within introns, of short fragments that probably affected or hindered their activity. This probably contributed to the accumulation of introns within the mt-genomes (Deng et al., 2018).

The circular mt-genome of the ectomycorrhizal fungus *Cantharellus* (*Agaricomycetes*) is another example of conservation and plasticity. It contains 15 protein-coding genes that are variable in length and GC content probably because they are under a purifying selection (Li et al., 2018b). Furthermore, rearrangements occurred between repetitive sequences and because of this tRNAs changed their positions. A similar process of purifying selection included repetitive sequences within 14 protein-coding genes in the mt-genome of *Hypsizygus marmoreus* (*Agaricomycetes*), another cellulolytic fungus, which probably was due to a positive selection that leads to a faster rate of evolution (Wang et al., 2019). Another example of conservation and plasticity was observed within the mt-genome of *Fusarium oxysporum* species complex (Hypocreales), which includes plant pathogens causing vascular wilts, rots, and damping-off on a broad range of crops. Within phylogenetically related isolates of the *F. oxysporum* species complex, mt-genomes undergo recombination which suggests that a parasexual cycle may exist (Brankovics et al., 2017). Regarding diversification of mt-genomes, Li et al. (2018c) showed that *Pleurotus citrinopileatus* and *Pleurotus platypus* (Agaricales) presented different numbers and sequences of introns, indicating that evolutionary events most probably include gene rearrangements and inversions. Also, *Ganoderma* and *Lyophyllum* (*Agaricomycetes*) had rearranged mt-genomes (Li et al., 2019a, b). Mobile elements play a crucial role in building the mt-genome architecture, but reshuffling appears to be characteristic of an organism or a group of them.

Researchers unsuccessfully tried to relate the size and structure of fungal mt-genomes with the organisms environmental requirements. Reductions in their size and/or complexity are hallmarks of pathogenic or mutualistic symbiotic relationships (Moran and Bennett, 2014). Pogoda et al. (2018) compared the mt-genome of 22 lichenized fungi with 167 mt-genomes of non-lichenized ones and found that the former ones lost the *atp9* gene. Simon et al. (2017) also found that the mt-genomes of the lichenized fungi *Ricasolia amplissima* and *Peltigera* sp. were identical regarding protein-coding genes but they were rearranged. The mt-genome of *Paramicrosporidium saccamoebae* (Rozellomycota), an intranuclear parasite of amoeba, has a gene content similar to Macrosporidia, suggesting that evolution in both groups was affected by repeated and independent gene losses, either due to variations in their parasitic strategies (e.g., host and subcellular localization) or to multiple transitions to parasitism (Quandt et al., 2017; Melnikov et al., 2018). On the other hand, within Mycosphaerellaceae (Capnodiales, Ascomycota), a family that includes many plant pathogens of economically important crops, mt-genomes are significantly expanded at least due to five independent intron insertions within genes of the electron transport chain (Arcilla et al., 2019). As a result of this, the mt-genome of closely related organisms is highly variable in size and gene order and contain truncated extra gene copies as well as accessory genes. The mt-genome of *S. lycopersici* and *Pyrenophora tritici-repentis*, two necrotrophic fungal plant pathogens that belong to the order Pleosporales, harbor 12 and 13 genes, respectively. Unlike the mt-genome of other fungi, they also have the *atp8* and *atp9* genes within the nuclear genome (Franco et al., 2017; Moolhuijzen et al., 2018). The mt-genomes of these fungi regarding the order, number, and gene orientation suggest they are plastic, which seems to be due at least in part to the action of homing endonucleases as well as repetitive elements, which provides more pieces of evidences of the crucial role these elements play in shaping the mt-genome architecture.

The mt-genome of another member of the Pleosporales, *Coniothyrium glycines*, the causal agent of soybean red leaf blotch, contains 12 mt-genes and 32 introns with a high number of homing endonucleases that represent approximately 54.1% of the mt-genome. However, the gene order of *nad6*-*rnl*-*atp6*, is conserved among members of Pleosporales (Stone et al., 2018). Kolesnikova et al. (2019) analyzed species of the genus *Armillaria* (Agaricales) and found that the mt-genome varied in size (98,896–122,167 bp) and arrangement. So, mobile genetic elements as well as homing endonucleases seem to be shaping the mt-genome architecture (Kolesnikova et al., 2019). However, several controversial findings remain regarding mt-genome plasticity. The mt-genome of *Rhizopogon* (*Agaricomycetes*), varied in length and base composition of proteins, rRNA, and tRNA genes, apparently due to switches that occurred between the mt and nuclear genomes and frequent intron loss/gain events that occur along with evolution. Also, in the mt-genome of *R. vinicolor* intronic regions contributed to expansion (Li et al., 2019c). On the other hand, representatives of *Hygrophorus* (Agaricales) and *Russula* (Russulales) have a conserved and reduced mt-genome within only three group I introns (Li et al., 2019d). The mt-genome of *Nectria cinnabarina* (Hypocreales), a plant pathogenic fungus that provokes cankers on many tree species and coral spot, presented a highly conserved gene composition and order compared to phylogenetically related fungi though they differed in the quantity as well as tRNAs order (Wang et al., 2016). While the organization of the *Phomopsis longicolla* mt-genome resembles that of other *Sordariomycetes*, unlike other genomes it has rearrangements of both tRNA and protein-coding genes (Kouvelis et al., 2005; Koloniuk et al., 2016). Other fungal pathogens of plants, such as *Pyricularia grisea* (Sordariomycetidae) the rice blast fungus (Xia et al., 2000) and *Ceratocystis fagacearum* (Microascales) the oak wilt pathogen (Kurdyla et al., 1995) have mt-genomes with a lower level of diversity than nuclear genomes, which is consistent with the hypothesis that in these fungi the nuclear genomes evolved at a slower rate than the mt-genome. Therefore, even though no relationship can be made between the genome arrangement, conservation, and gene composition with the environment where organisms develop, the evolution of mt-genomes within these organisms seems to evolve at a faster rate.

## The Mitogenome, Its Role in Fungal Virulence and a Target of Several Fungicides

As a result of the widespread use of new generation sequencing technologies, high-quality genomes are available and this allowed researchers to study the molecular bases of fungal biology in terms of sexual reproduction, virulence, secondary metabolite production as well as to know their ability to detoxify antifungal compounds (Mac Aogáin et al., 2019). Mitochondrial functions are essential for eukaryotes, and in phytopathogenic fungi, they are particularly important during their interaction with the hosts, where frequently environments with low oxygen tension arise (She et al., 2013; Sun et al., 2013). Mitochondria play a key role in virulence since the expression of mt-genes regulates fungal growth, biofilm, and hyphal growth. In addition to this, mt-genomes play a crucial role in resistance to antimicrobial compounds (Sandor et al., 2018). All these functions make mitochondria a good target to control diseases. Plant pathogenic fungi use both mitochondrial as well as nuclear-encoded proteins to infect plant hosts and provoke disease (Olson and Stenlid, 2001) and also their genomes carry genes coding for mechanisms of resistance (Joseph-Horne and Hollomon, 2000; Cowen et al., 2015), which is probably the reason they are frequent targets of some fungicides. However, any molecule targeted to mitochondrial proteins possesses a threat to eukaryotic organisms that share the biochemical functions and complexity of the genome (Brown et al., 2012). Therefore, it would be interesting to analyze the effects that recombination within the mt-genome might have on fungal virulence and also on pathogenesis.

Genes related to mitochondria and their replication seem to play a key role in growth and virulence of fungal pathogens of plants. In *Pyricularia oryzae*, the imperfect fission of mitochondria affects conidiation, growth, and virulence, while other changes in mt-genes affect the development of infection structures, invasion, and pathogenicity as well (Patkar et al., 2012; Khan et al., 2015). The mitochondrial fatty acid metabolism occurs through enoyl-CoA hydratase (*Ech1*), another enzyme that can be targeted to control fungal pathogens. If this metabolic pathway is blocked, the integrity as well as the morphology of the mitochondria is modified, and therefore, growth as well as, pathogenicity of the fungus, are affected (Mahlert et al., 2009). In this sense Mahlert et al. (2009) found that the loss of mitochondrial fission, which was related to a dynamin-related GTPase (*Dnm1*) gene, provoked a reduction in pathogenicity of *Ustilago maydis*, a maize pathogen (Mahlert et al., 2009). So, integrity as well as the functionality of the mt-genome are crucial for pathogens.

Virulence as well as host range of fungal plant pathogens also are affected by the architecture, arrangements, and modifications of the mt-genome. Populations of multilocus haplotypes of *Mycosphaerella graminicola* adapted to bread wheat (*Triticum aestivum*) and durum wheat (*T. turgidum*, Zhan et al., 2004) differed in haplotypes predominance. It was hypothesized that the low level of mitochondrial diversity within representatives of this pathogen is due to a selective sweep that is under the influence of both the host and natural selection (Zhan et al., 2004). Further evidence of the mitochondrial role on virulence was provided by hypovirulent mutants of *Cryphonectria parasitica* that happened to have plasmid-like elements interrupted by self-splicing introns (Monteiro-Vitorello et al., 1995; Baidyaroy et al., 2011). Also, in the *Heterobasidium annosum* species-complex, the causal agent of root and butt rot in conifers, virulence is under the control of the mt-genome. Olson and Stenlid (2001) found that virulence of both heterokaryons, as well as homokaryons of this fungus belonging to S and P intersterile groups, is due to uniparental transmission of mitochondria rather than the nucleus, being specifically the mitochondria of P origin related to a virulent phenotype (Olson and Stenlid, 2001). van de Vossenberg et al. (2018) found that a shift in the mitochondrial haplotypes, triggered within isolates of a community of *S. endobioticum* obtained from semi-resistant potato cultivars, resulted in changes in virulence. On the other hand, Moolhuijzen et al. (2018) found that a mutant of the wheat pathogen *Pyrenophora tritici-repentis* isolated from Australia containing an intron within the *cytochrome b* gene, lack the fungicide resistance site mutation observed in North American isolates. The effect of mt-genome recombination on the development and maintenance of virulence in plant pathogen populations is mostly due to their uniparental inheritance that is characterized by non-random segregation (Griffiths, 1996; Basse, 2010). Recently, it has been demonstrated that in *Fusarium zanthoxyli* and *F. continuum*, two canker-inducing pathogens of prickly ash (*Zanthoxylum bungeanum*), mitochondria only were maternally inherited (Zhou et al., 2019). Based on the effect of mt-genomes on virulence, their evolution should have a considerable impact on the epidemiology of diseases. Therefore, studies should be aimed at analyzing mt-genomes of pathogens populations to have a better understanding of mt-genes on virulence.

Due to the key role mitochondria play in microorganisms (Deacon, 2013), they have been and probably will be targeted by actual and future fungicides to prevent plant or cure plant diseases. The respiratory chain that leads to the synthesis of ATP has been a site targeted by fungicides, like compounds that uncouple the redox process and proton gradient from ATPases like carboxin and cyanide (I-V; Schuler and Casida, 2001). Many phytopathogenic fungi grow under an ample range of oxygen tensions that occur in diseased tissue, though necrotic tissues generate an extremely hostile environment for pathogens, since toxic compounds are released, which additionally might be accompanied by a reduction in nutrients. Alternative redox centers have been found in plant pathogenic fungi and their functions probably are associated with the adverse environmental conditions (Joseph-Horne and Hollomon, 2000). Because of this, theses complexes are alternative pathways to avoid the inhibitory activity of fungicides targeting respiratory redox centers.

Respiratory inhibitors that have been used as insecticides and miticides for more than 150 years were primarily targeted to the NAD(P)H dehydrogenase enzyme, known as complex I (Schuler and Casida, 2001). Piericidin A, bullatacin, thiangazole, and rotenone are natural products that block complex I, however, none of them have been released to the market. The explanation of this might be that alternative isozymes with NAD(P)H dehydrogenase activity were found in many fungal species (Joseph-Horne and Hollomon, 2000) and might therefore, provide an alternative pathway to evade the action of the fungicides that block complex I.

Complex II, which is known as succinate dehydrogenase or succinate-coenzyme Q reductase, also is the target of fungicides used against pathogens like *R. solani*, *Verticillium albo-atrum*, *U. maydis* and *Neurospora crassa* worldwide (Ragsdale and Sisler, 1970). An example of such compounds is carboxin that blocks the binding sites to ubiquinone, suppressing the citric acid cycle, inhibiting in this way fungal respiration by uncoupling the electron transport chain (Lyr, 1995).

Complex III, known as cytochrome *bc*, also is a target of fungicides like strobilurins that are known as the Qo inhibitors or QoIs (Becker et al., 1981). The latter ones were first introduced to the market in 1996 (Gisi et al., 2002). However, the development of resistance to strobilurins occurred in many different plant pathogens including fungi like, *Blumeria graminis* f. sp. *tritici*, *Mycosphaerella fijiensis*, and *Venturia inaequalis* as well as the oomycete *Plasmopara viticola* (Fraaije et al., 2002; Balba, 2007). Such resistance is related to a mutation that occurred within the coding sequence of cytochrome b (*cob*), where a G143 glycine is substituted by an alanine. If phenylalanine is replaced by leucine at position 129, F129L, the level of resistance is reduced, compared to the G143A substitution (Grasso et al., 2006). In addition to this, it has been stated that evolution of resistance to QoI fungicides based on G143A is not likely to occur in pathogens carrying an intron directly after this codon since this probably leads to a lethal phenotype (Grasso et al., 2006; Fisher and Meunier, 2008). The increased resistance of plant pathogens to such fungicides is not only a problem for agriculture, but also suggests the importance of studying if the evolution of mt-genomes is under other influences than the evolutionary clock, like anthropogenic ones. Additionally, synthetic fungicides have been developed based on QoIs compounds like kresoximmethyl, azoxystrobin and other derivatives that were commercially successful, due to their exceptionally wide range of crop protection (Affourtit et al., 2000). But still, many fungi can express an alternative QH2-oxidase that bypasses complex III and IV and confer resistance to this type of blocking agent (Vanlerberghe and McIntosh, 1997). Metyltetraprole, the first member of a new generation of QoIs, has a stable efficiency both in greenhouses and in the fields, even in the presence of G143A resistant mutants (Matsuzaki et al., 2020).

Complex IV, or cytochrome *c* oxidase, is a heme/copper terminal oxidase that uses cytochrome *c* as an electron donor. Inhibitors of complex IV are heme-binding inhibitors like azide, cyanide and sulfide, and inhibitors that compete with oxygen such as CO and NO, as well as polycations that compete with cytochrome c and phosphate or alkaline pH (Wallace and Starkov, 2000). Besides, heme is an important cofactor of enzymes such as sterol 14-α-demethylase, which is a target of azoles (e.g., fluconazole, ketoconazole, or vorizonazole). This prosthetic group is partially synthesized in mitochondria and plays a key role in several enzymes involved in sterol biosynthesis, respiratory chain, and cytochrome P450 synthesis and several functions are impacted by changes in heme cellular concentration (Bossche et al., 1995; Francois et al., 2005). Fe-S coupling in mitochondria can also be a target to control phytopathogenic fungi, since compounds such as auranofin reacts with thiol and selenol protein groups (Sannella et al., 2008), which blocks mitochondrial processes, such as Mia 40 and the cytochrome *c* oxidase biogenesis factor, both essential proteins for mitochondrial activities (Thangamani et al., 2017).

The complex V is composed by the H^+^-ATP synthetase that synthetizes ATP using the proton potential (Δμ_*H*__+_). This complex is inhibited by several mycotoxins like aurovertins A-E, leucinostatins A and B, venturicidin, and by oligomycins A-D and others. Furthermore, compounds such as flavonoids, the beta-adrenergic receptor antagonist propanolol, some anesthesics, the herbicide paraquat, several pyrethroid insecticides, DDT and parathion, several cationic dyes and other compounds are also blocking agents of this complex (Wallace and Starkov, 2000), which also is targeted by carbodiimide that is formed during bioactivation of the thiourea insecticide diafenthiuron (Schuler and Casida, 2001).

The mt-genome impact on virulence and pathogenicity also is under the influence of the environment that causes epigenetic modifications. In a recent study, Kang et al. (2017) characterized the mt-genome of Ophocordiceps sinensis and showed that usually affect the enzymatic activity of oxidative phosphorilationn and then the respiratory rate.

Repetitive mobile elements within fungal mt-genomes like palindromic sequences, AT-rich intergenic spacers, MUSEs, introns associated with mt-genome defects, rearrangements and genetic diversity of sequences flanking introns as well as epigenetic modifications suggest that mt-genomes also are prone to mutate in response to fungicides, leading this to the development of resistance to such molecules. It would be interesting to analyze the effects of recombination as well as all the other causes of changes within the mt-genome on fungal virulence and pathogenesis.

Last but not least is the fungal pathogen control on inhibition of essential matrix proteins, i.e., the mitochondrial processing peptidase (MPP) that is a nuclear enzyme involved in mitochondrial protein import (Braun and Schmitz, 1997), turning this protein into a possible target for fungicides. Thus, there are very many different ways for the mt-genome of pathogenic organisms like fungi to be altered or modified, though much remains to be studied regarding the effect of such changes on biological responses.

## Conclusion and Perspectives

Data regarding the structural organization of mt-fungal genomes, reveal the importance of mitochondria and their activity in several different essential metabolic processes, however, they are plastic and dynamic but they still are used in studies of evolution within the kingdom fungi. Several processes occurring within mitochondria lead to a size reduction in their genomes.

It can be concluded that mt-genomes are plastic but still conserved which is good in terms of functionality, and in addition to this they harbor DNA repair mechanisms. Their relatively small size, circular-mapping topology, and multi-copy nature of the mt-DNA simplify sequencing and assembly, this enables researchers to make a robust comparative analysis to study evolution. Even though mobile elements play a crucial role in building the mt-genome architecture, reshuffling appears to be characteristic of an organism or a group of them. The degree of gene conservation and ordination within fungal mt-genome seemed to be dependent upon the fungal group studied. The mt-genomes of plant pathogenic fungi regarding order, number and gene orientation suggest they are plastic in part due to the action of homing endonucleases as well as repetitive elements, which provides more evidences of the crucial role these elements play in shaping the mt-genome architecture. Mt-genomes are prone to mutations due to their richness in repetitive mobile elements, AT-rich intergenic spacers, MUSEs, introns, rearrangements, and genetic diversity of sequences flanking introns. In consequence, this could occasionally lead to the onset of adaptive mutants that are resistant to fungicide.

Since the degree of conservation and ordination of genes vary according to the group studied, a preliminary analysis should be done in order to select “*a priori*” reliable phylogenetic markers. Among mt-genomic features, those involved at increasing the synthesis of iron-sulfur clusters and heme cofactors are probably the most important ways, considering the role S and Fe play on fungal pathogenesis and other biological processes as well. However, information regarding the rate of mt-genome mutations is lacking as well as its impact on functions, maintenance, and evolution of these genomes.

Fungal phytopathogens mt-genomes also are related to virulence. Although the mt-genome of model phytopathogens have been structurally characterized it is crucial to increase their number in databases to establish if there is any particular pattern that might explain fungal virulence and pathogenesis. The mitochondrial mechanisms of mt-DNA maintenance and/or repair should be deepened further in order to better understand their biological significance and to develop new strategies to manage the threat of phytopathogenic fungi. A more profound understanding of fungal mt-genome regarding structure and function should provide the tools to manage phytopathogenic fungi and their virulence.

## Author Contributions

All authors contributed on the revision and conceptualization of this manuscript. RM, MS, and PB elaborated the manuscript. RM and MF made the figures.

## Conflict of Interest

The authors declare that the research was conducted in the absence of any commercial or financial relationships that could be construed as a potential conflict of interest.
